# Response of Bacterial and Fungal Soil Communities to Chinese Fir (*Cunninghamia lanceolate*) Long-Term Monoculture Plantations

**DOI:** 10.3389/fmicb.2020.00181

**Published:** 2020-02-28

**Authors:** Xian Liu, Yuzhe Wang, Yuhui Liu, Hui Chen, Yalin Hu

**Affiliations:** ^1^Forest Ecology and Stable Isotope Research Center, College of Forestry, Fujian Agriculture and Forestry University, Fuzhou, China; ^2^Xinkou Forest Farm, Fujian Agriculture and Forestry University, Sanming, China

**Keywords:** successive plantation, bacterial community, fungal community, Ion S5^TM^ XL sequencing platform, Chinese fir plantation

## Abstract

Successive rotation and monoculture, as common silvicultural practices, are extensively applied worldwide, particularly in subtropical Chinese fir (*Cunninghamia lanceolata*) plantations in southern China. Although regeneration failure and productivity decline are frequently observed in continuous monoculture plantations, the potential mechanisms are still unclear. In this study, high-throughput sequencing was used to compare the diversity and composition of bacterial and fungal communities among different generations of Chinese fir plantation (first rotation, FRP; second rotation, SRP; third rotation, TRP) and natural forest (NF) in December and June. Our results showed significant declines in richness and diversity of bacterial and fungal communities in TRP compared with FRP and SRP, but no significant difference between FRP and SRP. The fungal phyla with high relative abundance were *Basidiomycota* (12.9–76.9%) and *Ascomycota* (14.3–52.8%), while the bacterial phyla with high relative abundance were *Acidobacteria* (39.1–57.7%) and *Proteobacteria* (21.2–39.5%) in all treatments at both sampling months. On average, the relative abundance of *Basidiomycota* in TRP increased by 53.4%, while that of *Ascomycota* decreased by 37.1% compared with FRP and SRP. Moreover, soil NH_4_^+^–N, pH, and DOC appear to be the key factors in shaping the fungal communities, while soil NH_4_^+^–N, DOCN, and AP primarily drive the changes in bacterial communities. Collectively, our findings highlighted the alteration of soil bacterial and fungal communities induced by changes in soil nutrient environment in different generations of continuously cultivated Chinese fir plantation.

## Introduction

Chinese fir [*Cunninghamia lanceolata* (Lamb.) Hook.] is one of the most important plantations in subtropical China due to its high yield and good timber quality. At present, Chinese fir plantation covers over 12 × 10^6^ ha in China, accounting for approximately 24 and 6.5% of all forest plantation area in China and worldwide, respectively (FAO, 2006; Chen G.S. et al., 2013). Since the 1950s, the area of Chinese fir plantation has been greatly enlarged, in order to meet the rising demand for timber caused by economic development and population increase in China. However, the current silvicultural practices for Chinese fir, including monoculture, continuous plantation, and shortened rotation time of 20 years (Wang et al., 2005), have led to multiple ecological problems such as soil nutrient depletion and timber productivity decline (Ma et al., 2007; Liu et al., 2010; Tian et al., 2011). The reasons that are responsible for yield decline are soil nutrient deficiency (Yang et al., 2004; Zhou et al., 2015), autotoxicity of root exudates (Huang et al., 2000; Lin et al., 2008), and alterations in soil microbial community (Wu et al., 2017). However, comprehensive and in-depth study into soil microbial community and the mechanisms involved in soil–microbe interactions in Chinese fir plantations is still lacking.

Worldwide, successive rotation as a common silvicultural practice has been extensively studied, but the results were far from universal. For example, no productivity decline was found between the second and third rotations of *Pinus patula* plantations in Switzerland (Evans, 1999), and plantation yield even increased over successive rotations in the Amazon basin of Brazil because of evolution of silvicultural practices and genetic improvement (McNabb and Wadouski, 1999). Recent studies showed that biological processes rather than external nutrient acquisition played a dominant role in nutrient cycling in Chinese fir plantations with the increase in stand age (Ma et al., 2007; Wu et al., 2017). With the rapid development of large-scale high-throughput sequencing system, it became possible to evaluate the variations on microbial communities among different generations of Chinese fir plantation.

Soil microbial communities are pivotal drivers of soil nutrient cycling processes (e.g. litter decomposition, mineralization), thus maintaining the nutrient balance in forest ecosystems (Moller et al., 1999; Liu et al., 2010; Bennett et al., 2012). Besides, soil microorganisms, themselves, also act as sources and sinks of active soil nutrients (Smith and Paul, 1990). As a result, microbes are highly sensitive to the changes in soil environment and nutrient availability (He et al., 2017; Wang et al., 2018), and microbial responses are widely used as ecological indicators for soil quality (Bending et al., 2004). Successive plantings of *C. lanceolata* plantations were reported to decrease soil pH, organic matter content, C/N ratio, available N and P (Yang et al., 2004; Selvaraj et al., 2017; Wu et al., 2017), which was likely to modify the composition and diversity of soil microbial communities. Nevertheless, previous investigations on soil microbial composition in different planting generations of Chinese fir were conducted by the methods of phospholipid fatty acid analysis (PLFA) and community-level physiological profiles (CLPP) (Wu et al., 2017). These technologies are greatly limited with regard to the detecting resolution on taxonomic level, thus failing to provide in-depth knowledge on microbial communities involved in successive rotations of Chinese fir plantation.

Overall, soil microbial communities are affected by a combination of factors, such as climate (Zhang et al., 2016), soil nutrient status (DeForest and Scott, 2010), plantation age (Chen G.S. et al., 2013), site preparation and harvest practices (Hartmann et al., 2012), geographical location (Liao et al., 2015), and so on. Furthermore, seasonal shifts also influence soil microbial community composition via affecting the soil physiochemical characteristics (Voříšková et al., 2014; Wu et al., 2017). Owing to these confounding factors, contradictory results were reported on the impact of successive rotation on soil microbial community structure. For example, Chen F.L. et al. (2013) reported a decreased soil microbial community size (measured by PLFA abundance) and enzymatic activities in successively planted *Eucalyptus*, but this degeneration was greatly alleviated by the increase in stand age; Wu et al. (2017) reported that soil microbial community diversity significantly decreased in successive rotations of Chinese fir plantations. However, an increased soil fungal diversity during successive rotations of *Casuarina equisetifolia* plantations was reported by Zhou et al. (2019). Therefore, it still remains unclear how bacterial and fungal communities are affected during successive rotations in Chinese fir monoculture, particularly based on high-throughput technology.

This study employed high-throughput sequencing methods to examine the changes in soil bacterial and fungal community structures among different generations of Chinese fir plantation (FRP, SRP, TRP) and natural forest (NF), aiming to elucidate the major environmental factors driving the changes in soil microbial communities. Specifically, we hypothesized that (1) continuous planting of *C. lanceolata* plantations would decrease soil bacterial and fungal community diversity and modify the soil microbial community structure in different planting generations; (2) the changes in the bacterial and fungal community structure would be related to soil nutrient availability (e.g. dissolved organic C and N, NH_4_^+^–N, NO_3_^–^–N) and/or environmental factors (e.g. soil pH, moisture). Our study will help facilitate a better understanding of the microbial diversity and community structure in response to successive rotations, as well as provide a theoretical basis for developing more sustainable management practices in a forest ecosystem.

## Materials and Methods

### Study Site and Soil Sampling

This study was conducted at the Xinkou Forest Farm of Fujian Agriculture and Forestry University (26°10′E, 117°28′N) at Sanming, Fujian Province in southeast China. The climate is subtropical humid monsoon, with an annual precipitation of 1,749 mm and a mean annual temperature of 19.1°C. The mean evapotranspiration is 1,585 mm, and the annual mean relative humidity is 81.0%. The altitude of this region ranges from 175 to 264 m above sea level. The soil is dominated by Silty Oxisol based on the United States Department of Agriculture (USDA) soil taxonomy, which was derived from parent sandstone and shale (Zhou et al., 2015).

“Space-for-time substitution” method was used to investigate the changes in soil microbial communities among rotations of Chinese fir plantations, since the rotation period for Chinese fir is generally 20–25 years (Tian et al., 2011). The selected three Chinese fir plantations were all at a mature stage (>26 years). The first Chinese fir rotation plantation (FRP) was established in 1973 after clearcutting a broad-leaved forest, and the second rotation plantation (SRP) was established in 1990 in a cutover site of a first-generation Chinese fir plantation, and the third rotation plantation (TRP) was established in 1979 after two rotations of Chinese fir plantation at the same site. Moreover, one natural forest (*Castanopsis kawakamii*; NF) was selected as the reference site. In 2017, four experimental plots (20 m × 20 m each) were established in each plantation, with a buffer zone >1 km among each plot, giving a total of 16 plots in this study.

Soil samples were taken in December 2017 and June 2018, and in total, 32 soil samples were collected. For each sampling, six cores of surface soil samples (0–10 cm) were randomly collected using a 3.8-cm-diameter auger and mixed into one composite soil sample for each plot. Soil samples were stored at field moisture content in plastic bags at 4°C after removing visible matter, such as loose gravel and plant debris. Fresh soils were homogenized and sieved to ≤2 mm and divided into two portions. One portion was stored at −80°C for molecular analysis, and another portion was stored at 4°C for soil chemical analysis.

### Soil Analysis

Soil pH was measured with a pH meter using a soil-to-water ratio of 1:5. Soil moisture was determined gravimetrically after drying the soil at 105°C for 24 h, and all results were expressed on an oven-dry basis. Microbial biomass C (MBC) and N (MBN) were determined by the chloroform fumigation extraction method (Vance et al., 1987; Jenkinson, 1988). Soil mineral N (NH_4_^+^–N, NO_3_^–^–N) was extracted using 2 M KCl and measured by Westco Smart Chem Discrete Wet Chemistry Analyzer (Westco Scientific Instruments, United States). Total C (TC) and N (TN) were measured using an elemental analyzer (Vario MICRO cube, Elementar, Germany). Dissolved organic C (DOC) and N (DON) were extracted by 2 M KCl using a soil-to-water ratio of 1:5 and then measured by a TOC-VCPH/CPN analyzer fitted with a TN unit (Shimadzu Scientific Instruments, Japan). Soil available P (AP) was extracted by 0.5 M NaHCO_3_ solution, colored by a molybdenum–antimony solution, and measured using the spectrophotometric method.

### DNA Extraction and High-Throughput Sequencing

Genomic DNA was extracted from 0.3 g of each composite soil sample from each sample plot (*n* = 16), using the MoBio Powersoil^TM^ DNA Isolation Kit (MO BIO Laboratories, Carlsbad, CA, United States) according to the manufacturer’s instructions, with the final elution step in deionized water instead of TE buffer. DNA quantity and concentration were determined using a DeNovix DS-11 spectrophotometer (DeNovix, Wilmington, DE, United States).

The V3–V4 region of the bacterial 16S *r*RNA gene and ITS1 region of the fungal internal transcribed spacer (ITS) were amplified using the bacterial-specific primer of 341F and 806R, eukaryotic-specific primers ITS5-1737F and ITS2-2043R, with unique barcodes added to the reverse primer, respectively. All PCR reactions were carried out in 30-μL reactions with 15 μL of Phusion^®^ High-Fidelity PCR Master Mix (New England Biolabs, United Kingdom), 0.2 μM of forward and reverse primers, and 10 ng of template DNA. The thermal cycling conditions consisted of initial denaturation at 98°C for 1 min, followed by 30 cycles of denaturation at 98°C for 10 s, annealing at 50°C for 30 s, and elongation at 72°C for 30 s, and then a final extension step at 72°C for 5 min. Amplified products were detected by electrophoresis in a 2% (w/v) agarose gel and purified with QIAquick PCR Purification Kit (QIAGEN, Germany). Sequencing libraries were generated using the Ion Plus Fragment Library Kit 48 rxns (Thermo Scientific) following the manufacturer’s recommendations. The library quality was assessed on the Qubit^®^ 2.0 Fluorometer (Invitrogen, United States). The high-throughput sequencing was conducted on the Ion S5^TM^ XL platform at Novogene Bioinformatics Technology Co., Ltd. (Beijing, China), and 400-bp/600-bp single-end reads were generated.

### Bioinformatics Analysis

Single-end reads were assigned to samples based on their unique barcode and truncated by cutting off the barcode and primer sequence. Quality filtering on the raw reads were performed under specific filtering conditions to obtain the high-quality clean reads according to the Cutadapt (Martin, 2011) (V1.9.1)^1^ quality-controlled process. The reads were compared with the reference database using the UCHIME algorithm (UCHIME Algorithm)^2^ to detect chimera sequences, and then the chimera sequences were removed (Edgar et al., 2011; Haas et al., 2011). Then the clean reads were finally obtained. Sequence analyses were performed by the Uparse software (Edgar, 2013)^3^. Sequences with ≥97% similarity were assigned to the same OTUs. The SILVA ribosomal RNA gene database was used for sequence/OTU classification. All the sequencing data have been deposited in the European Nucleotide Archive under accession number PRJEB29419. Observed OTU numbers, Chao1 richness, ACE evenness, as well as Shannon’s diversity indices, were calculated as metrics for alpha diversity. Beta diversity was calculated based on both weighted and unweighted UniFrac by the QIIME software.

### Statistical Analysis

The normality of all data were checked and met before the statistical analysis. Two-way analysis of variance (ANOVA) with repeated measures was used to determine if there was a significant interaction between sampling time and plantation rotations. One-way ANOVA followed by LSD *post hoc* test was performed to evaluate the effect of successive rotations at each sampling time when there was significant interactions between sampling time and planting rotations. All the above statistical analyses were conducted with the SPSS 22 software (SPSS Inc., Chicago, IL, United States). Non-metric multidimensional scaling (NMDS) was generated from the Bray–Curtis distance to visualize the bacterial and fungal community structure among different plantation rotations using the R software. The relationship between soil microbial community composition and soil environmental factors (pH, TC, TN, TCN, DOC, DON, DOCN, NH_4_^+^–N, NO_3_^–^–N, AP) was analyzed by redundancy analysis (RDA) based on OTU relative abundance data.

## Results

### Soil Physiochemical Properties

Repeated measures ANOVA showed significant interactions between planting rotations and sampling time on soil properties such as moisture, TC, TN, NH_4_^+^–N, NO_3_^–^–N, and MBN (Supplementary Table S1) (*P* < 0.05). Different management practices (NF and successive rotations) significantly altered soil pH, moisture, TC, TN, DOC, NH_4_^+^–N, NO_3_^–^–N, and AP, while sampling time significantly affected all measured parameters except for soil C/N ratio (Supplementary Table S1) (*P* < 0.05). The pH ranged from 4.00 ± 0.03 to 4.42 ± 0.05 throughout the sampling period. Significant higher pH was observed in SRP compared with FRP and TRP, and no significant difference was observed between FRP and TRP at both sampling times (Table 1) (*P* < 0.05). In December, the SRP had significant higher soil moisture compared with FRP and TRP, while no significant difference was found between FRP and TRP (Table 1) (*P* < 0.05). In June, there was no significant difference in soil water content among FRP, SRP, and TRP (Table 1) (*P* > 0.05). At both sampling times, no significant difference was observed in soil NH_4_^+^–N, DON, DOCN, MBC, MBN, and MBCN among FRP, SRP, and TRP. In June, significant higher TC, TN, and TCN were found in TRP compared with those in FRP, while no significant difference was found between SRP and FRP, and SRP and TRP. At both sampling times, significant lower AP was found in TRP compared with those in NF, FRP, and SRP.

**TABLE 1:** Soil physical and chemical characteristics in different generations of Chinese fir plantation (December and June) at the Xinkou Forest Farm, Fujian, China.

**Season**	**Treatment**	**pH**	**Moisture (%)**	**TC (g⋅kg^–1^)**	**TN (g⋅kg^–1^)**	**TCN**	**NH_4_^+^–N** (mg⋅kg^–1^)	**NO_3_^–^–N (mg⋅kg^–1^)**	**DOC (mg⋅kg^–1^)**
December	NF	4.30 ± 0.02^a^	17.06 ± 0.65^c^	15.83 ± 1.59^b^	1.13 ± 0.15^b^	14.20 ± 0.77^a^	30.69 ± 5.05^a^	0.76 ± 0.13^b^	122.32 ± 10.41^b^
	FRP	4.00 ± 0.03^c^	21.43 ± 0.45^b^	22.45 ± 2.18^a^	1.54 ± 0.09^a^	14.47 ± 0.57^a^	5.81 ± 0.28^b^	4.96 ± 1.00a	215.99 ± 39.70^a^
	SRP	4.19 ± 0.03^b^	27.46 ± 1.23^a^	25.58 ± 1.79^a^	1.77 ± 0.04^a^	14.43 ± 0.76^a^	6.38 ± 0.68^b^	4.57 ± 0.48^a^	221.06 ± 9.11^a^
	TRP	4.07 ± 0.03^c^	20.51 ± 1.13^b^	27.15 ± 1.66^a^	1.75 ± 0.12^a^	15.53 ± 0.24^a^	7.97 ± 1.00^b^	4.81 ± 0.44^a^	159.23 ± 8.57^ab^
June	NF	4.42 ± 0.05^a^	28.41 ± 1.62^b^	28.60 ± 2.25a	1.81 ± 0.07^ab^	15.71 ± 0.65^a^	6.64 ± 1.47^a^	8.61 ± 1.39^b^	88.29 ± 3.55^b^
	FRP	4.23 ± 0.02^c^	29.05 ± 0.28^ab^	21.72 ± 0.91^b^	1.54 ± 0.04^b^	14.12 ± 0.27^b^	5.69 ± 2.59^a^	13.48 ± 1.00^a^	134.34 ± 6.27^a^
	SRP	4.26 ± 0.03^b^	32.78 ± 2.04^a^	26.00 ± 1.79^ab^	1.78 ± 0.09^ab^	14.56 ± 0.48^ab^	7.40 ± 0.18^a^	2.97 ± 0.57^c^	89.22 ± 7.30^b^
	TRP	4.14 ± 0.04^c^	31.06 ± 0.64^ab^	29.82 ± 2.22^a^	1.88 ± 0.15^a^	15.95 ± 0.49^a^	3.16 ± 0.35^a^	10.57 ± 0.48b	117.60 ± 6.99^a^

**Season**	**Treatment**	**DON (mg⋅kg^–1^)**	**DOCN**	**MBC (mg⋅kg^–1^)**	**MBN (mg⋅kg-^1^)**	**MBCN**	**AP (mg⋅kg^–1^)**		

December	NF	11.77 ± 1.11^a^	10.53 ± 0.96^c^	272.66 ± 19.99^ab^	39.28 ± 1.98^a^	6.95 ± 0.37^a^	5.20 ± 0.17^b^		
	FRP	14.44 ± 1.38^a^	14.84 ± 1.74^ab^	257.63 ± 53.90^c^	36.19 ± 9.56^a^	7.38 ± 0.75^a^	5.77 ± 0.16^a^		
	SRP	14.97 ± 0.96^a^	14.93 ± 1.08^bc^	307.37 ± 20.45^bc^	41.95 ± 0.55^a^	7.33 ± 0.49^a^	5.12 ± 0.16^b^		
	TRP	12.03 ± 1.52^a^	14.01 ± 2.29^bc^	207.81 ± 16.83^bc^	27.03 ± 6.59^a^	8.57 ± 1.33^a^	5.28 ± 0.17^b^		
June	NF	21.68 ± 0.79^a^	4.07 ± 0.07^c^	497.30 ± 24.25^ab^	95.31 ± 5.87^a^	5.23 ± 0.11^a^	2.44 ± 0.11^b^		
	FRP	23.07 ± 0.65^a^	5.81 ± 0.14^ab^	281.77 ± 28.73^c^	45.64 ± 6.19^b^	6.33 ± 0.60^a^	2.72 ± 0.07^a^		
	SRP	20.90 ± 1.58^a^	4.28 ± 0.22^bc^	403.42 ± 75.95^bc^	69.17 ± 8.90^ab^	5.75 ± 0.42^a^	2.59 ± 0.15^b^		
	TRP	24.34 ± 1.99^a^	4.93 ± 0.52^bc^	412.58 ± 60.44^bc^	69.74 ± 13.81^ab^	6.28 ± 0.75^a^	2.48 ± 0.08^b^		

*TC, total carbon; TN, total nitrogen; TCN, TC/TN ratio; DOC, dissolved organic carbon; DON, dissolved organic nitrogen; DOCN, DOC/DON ratio; MBC, microbial biomass carbon; MBN, microbial biomass nitrogen; MBCN, MBC/MBN ratio; AP, available phosphorus; NF, natural forest; FRP, first rotation plantation; SRP, second rotation plantation; TRP, third rotation plantation. Values are the means ± standard errors (n = 4). Different letters indicate significant differences among the treatments (*P* < 0.05).*

### Soil Microbial Community Diversity

After quality filtering and chimera check, a total of 2,509,952 and 2,565,913 high-quality 16S *r*RNA/ITS gene sequences were obtained with an average length of 413 and 237 across all samples, respectively. The number of 16S *r*RNA sequences obtained per sample varied from 55,856 to 92,288, and the number of ITS gene sequences per sample varied from 80,017 to 80,411 (Supplementary Table S2). These sequences were grouped into OTU (97% similarity), and the number of bacterial and fungal OTU ranged from 1,044 to 1,606, 1,109 to 1,544, respectively, depending on different soil samples (Supplementary Table S2).

Repeated measures ANOVA showed no interaction between planting rotations and sampling time on soil fungal and bacterial diversity indices such as OTUs, Chao1, and ACE (Supplementary Table S3). At both sampling months, the number of bacterial OTUs, ACE, Chao1, and Shannon index significantly decreased in TPR compared with those in NF, FRP, and SRP (Table 2) (*P* < 0.05). On the other hand, the number of fungal OTUs and Shannon index significantly increased in SRP compared with those in NF and TRP, while no significant change was observed between NF, FRP, and TRP, and between FRP and SRP at both sampling months (Table 3) (*P* < 0.05). The fungal ACE index was significantly lower in TRP than in SRP, while there was no significant difference among NF, FRP, and TRP at both sampling months (Table 3) (*P* < 0.05). The fungal Chao1 index did not change with planting rotations at both sampling months (Table 3) (*P* > 0.05).

**TABLE 2:** Soil bacterial alpha diversity indices in different generations of Chinese fir plantation (December and June) at the Xinkou Forest Farm, Fujian, China.

**Season**	**Treatment**	**OTUs**	**Richness**		**Diversity**
			**ACE**	**Chao1**	**Shannon**
December	NF	1363 ± 29^a^	1592 ± 56^a^	1579 ± 59^a^	8.15 ± 0.06^a^
	FRP	1350 ± 61^a^	1583 ± 100^a^	1575 ± 95^a^	8.08 ± 0.02^a^
	SRP	1384 ± 76^a^	1647 ± 129^a^	1662 ± 130^a^	8.08 ± 0.07^a^
	TRP	1193 ± 51^b^	1346 ± 82^b^	1315 ± 88^b^	7.50 ± 0.24^b^
June	NF	1258 ± 37^a^	1398 ± 39^a^	1393 ± 39^a^	8.01 ± 0.08^a^
	FRP	1299 ± 41^a^	1404 ± 45^a^	1399 ± 52^a^	8.15 ± 0.08^a^
	SRP	1313 ± 20^a^	1423 ± 26^a^	1409 ± 18^a^	8.11 ± 0.06^a^
	TRP	1240 ± 15^b^	1345 ± 19^b^	1332 ± 19^b^	8.07 ± 0.04^b^

*OTUs, operational taxonomic units (97% similarity). Values are the means ± standard errors (*n* = 4). Different letters indicated significant differences among the treatments (*P* < 0.05). NF, natural forest; FRP, first rotation plantation; SRP, second rotation plantation; TRP, third rotation plantation.*

**TABLE 3:** Soil fungal alpha diversity indices in December and June at the Xinkou Forest Farm, Fujian, China.

**Season**	**Treatment**	**OTUs**	**Richness**		**Diversity**
			**ACE**	**Chao1**	**Shannon**
December	NF	1325 ± 42^b^	1486 ± 41^ab^	1469 ± 43^a^	6.63 ± 0.20^b^
	FRP	1357 ± 107^ab^	1500 ± 107^ab^	1496 ± 108^a^	7.03 ± 0.37^ab^
	SRP	1430 ± 38^a^	1556 ± 42^a^	1547 ± 39^a^	7.59 ± 0.14^a^
	TRP	1171 ± 27^b^	1306 ± 19^b^	1297 ± 19^a^	6.30 ± 0.42^b^
June	NF	1189 ± 32^b^	1393 ± 23^ab^	1361 ± 27^a^	6.04 ± 0.34^b^
	FRP	1335 ± 79^ab^	1503 ± 69^ab^	1489 ± 70^a^	6.95 ± 0.44^ab^
	SRP	1386 ± 59^a^	1629 ± 167^a^	1953 ± 516^a^	7.22 ± 0.15^a^
	TRP	1315 ± 48^b^	1475 ± 33^b^	1451 ± 32^a^	6.65 ± 0.30^b^

*OTUs, operational taxonomic units (97% similarity). Values are the means ± standard errors (*n* = 4). Different letters indicated significant differences among the treatments (*P* < 0.05). NF, natural forest; FRP, first rotation plantation; SRP, second rotation plantation; TRP, third rotation plantation.*

### Soil Microbial Community Composition

**FIGURE 1:**
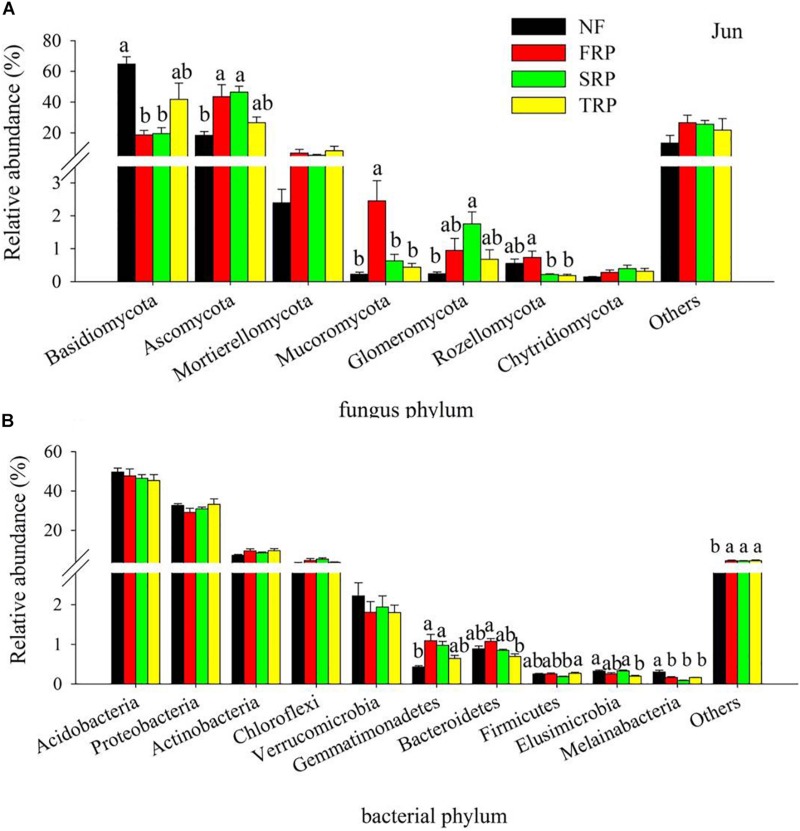
Relative abundance of the most abundant fungal **(A)** and bacterial **(B)** groups in different plantations in June at the phyla level. Others represented unclassified groups. Data are means ± standard error (*n* = 4). Significant differences are indicated with distinct letters. NF, natural forest; FRP, first rotation plantation; SRP, second rotation plantation; TRP, third rotation plantation.

The fungal phyla with high relative abundance were *Basidiomycota* (12.9–76.9%) and *Ascomycota* (14.3–52.8%) in all treatments at both sampling months, followed by *Mortierellomycota* (1.6–12.5%) (Figure 1A and Supplementary Figure S1a). Different plantation rotations had various impacts on fungal and bacterial compositions at the phylum level in December and June, respectively. In both months, the relative abundance of *Basidiomycota* significantly decreased in FRP and SRP relative to NF, while no significant difference was observed between NF and TRP (Figure 1A and Supplementary Figure S1a) (*P* < 0.05). In December, the relative abundance of *Ascomycota* significantly increased in FRP and SRP compared with NF, while no significant difference was observed between NF and TRP, as well as FRP and TRP (Supplementary Figure S1a) (*P* < 0.05). There was no significant difference in the relative abundance of phyla *Mortierellomycota*, *Glomeromycota*, *Rozellomycota*, and *Chytridiomycota* among the different plantation rotations (Supplementary Figure S1a) (*P* > 0.05). In June, The relative abundance of *Ascomycota* significantly increased in FRP and SRP compared with that of NF, while no significant difference was observed between NF and TRP, and among FRP, SRP, and TRP (Figure 1A) (*P* < 0.05). There was no significant difference in the relative abundance of *Mortierellomycota* and *Chytridiomycota* among different plantation rotations (Figure 1A) (*P* > 0.05).

The bacterial phyla with a high relative abundance were *Acidobacteria* (39.1–57.7%) and *Proteobacteria* (21.2–39.5%) in all treatments at both sampling months, followed by *Actinobacteria* (6.1–19.2%), *Chloroflexi* (2.0–9.3%), and *Verrucomicrobia* (0.5–4.3%) (Figure 1B and Supplementary Figure S1b). Overall, the relative abundance of *Acidobacteria*, *Proteobacteria*, *Actinobacteria*, *Chloroflexi*, and *Verrucomicrobia* were not influenced by age of successive rotations (Figure 1B and Supplementary Figure S1b) (*P* > 0.05).

**FIGURE 2:**
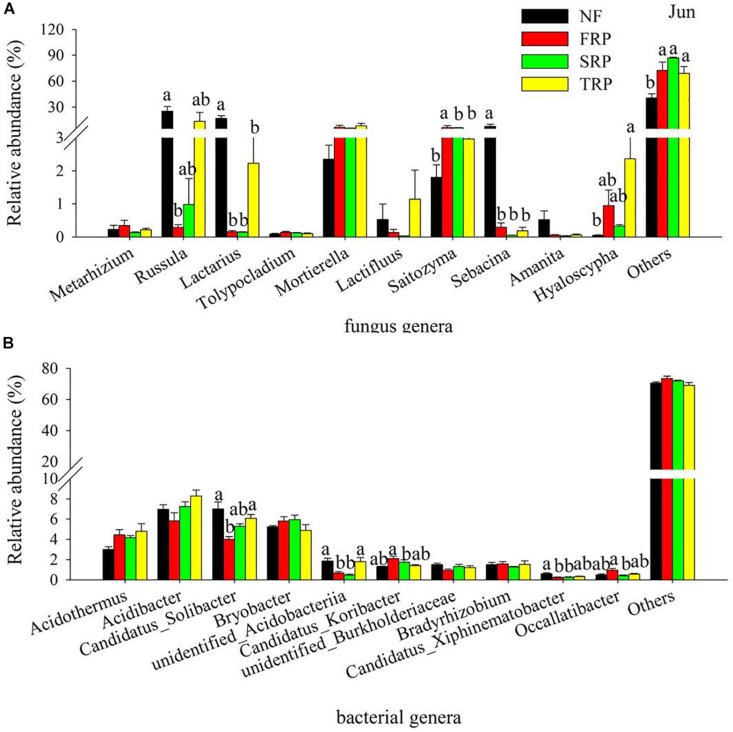
Relative abundance of the most abundant fungal **(A)** and bacterial **(B)** groups in different plantations in June at the genus level. Others represented unclassified groups. Data are means ± standard error (*n* = 4). Significant differences are indicated with distinct letters. NF, natural forest; FRP, first rotation plantation; SRP, second rotation plantation; TRP, third rotation plantation.

At the genus level, the most abundant species were largely unclassified, ranging from 65.8 to 76.3%, and 40.9 to 92.4% for bacteria and fungi, respectively (Supplementary Figure S2). The bacterial genus with a high relative abundance were *Acidothermus*, *Acidobacter*, *Candidatus solibacter*, and *Bryobacter* (Figure 2B and Supplementary Figure S2b), while the fungal genera with high relative abundance appeared to be inconsistent in different plantation rotations (Figure 2A and Supplementary Figure S2a).

**FIGURE 3:**
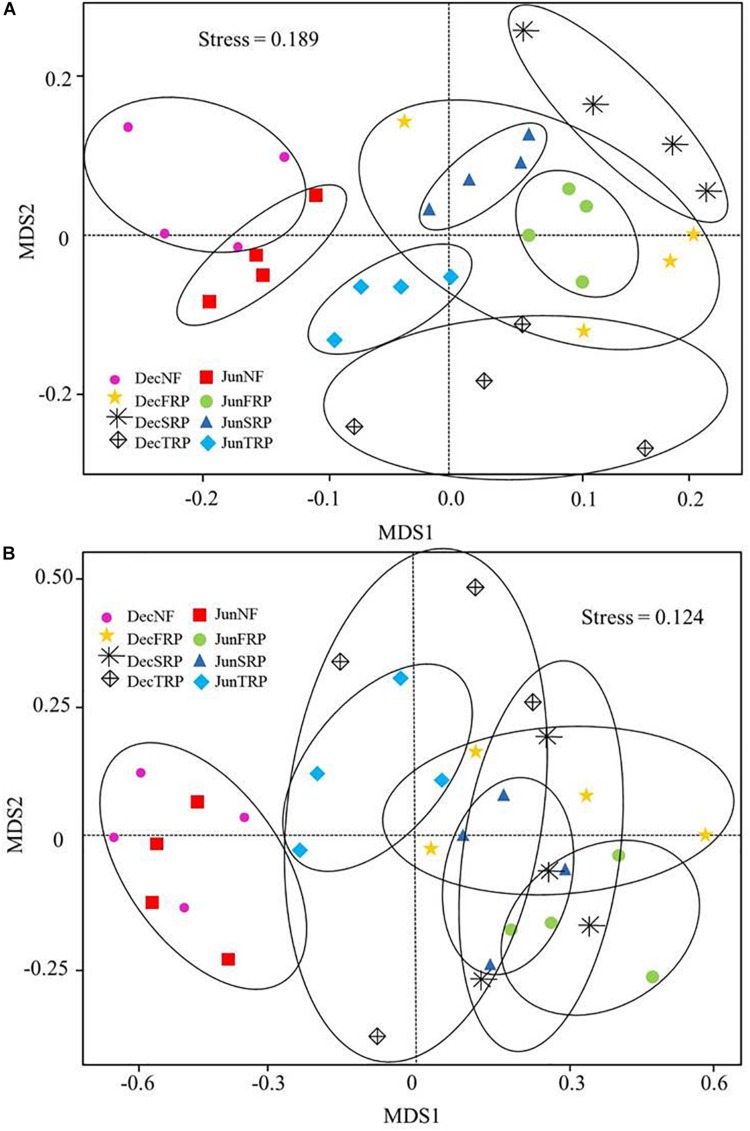
Non-metric multidimensional (NMDS) scaling plot of bacterial **(A)** and fungal **(B)** community structure across all soil samples in different plantations and sampling times (using combined December and June data). Stress value is indicated in the figure. Data points are from different plantations and sampling seasons (NF, natural forest; FRP, first rotation plantation; SRP, second rotation plantation; TRP, third rotation plantation; Dec, December; Jun, June).

The NMDS clearly grouped the bacterial and fungal communities according to the four plantations and sampling months (Figures 3A,B). Particularly, the bacterial and fungal communities were clearly separated between NF and successive rotations (FRP, SRP, and TRP) across two sampling months, and the fungal communities appeared to be clustered more closely compared to their bacterial counterpart (Figures 3A,B).

### Relationship Between Microbial Community Structure and Soil Environmental Factors

**FIGURE 4:**
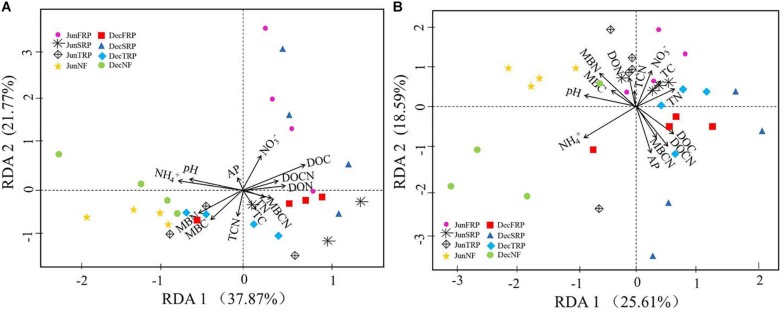
Redundancy analysis (RDA) illustrating the effects of environmental factors (arrows) on fungal **(A)** and bacterial **(B)** community structure (symbols) across all soil samples in different plantations and sampling times (using combined December and June data). The values of axes 1 and 2 are the percentage that can be explained by the corresponding axis. Data points are from different plantations and sampling seasons (NF, natural forest; FRP, first rotation plantation; SRP, second rotation plantation; TRP, third rotation plantation; Dec, December; Jun, June).

According to RDA eigenvalues, axes 1 and 2 accounted for 37.87 and 21.77% of the overall variance in soil fungal communities across two sampling months, respectively (Figure 4A). Moreover, RDA clearly revealed that soil ammonium, DOC, and pH were the most significant contributors to the variation of fungal communities (Figure 4A). There were significant positive relationships between soil MBC, MBN, and fungal diversity indices (OTUs, Chao1, ACE, Shannon index), and significant negative relationship between soil MBCN and Shannon index of soil fungal communities in both sampling months (Supplementary Table S4).

Similarly, the RDA showed that the first and second axes explained 25.61 and 18.59%, respectively, of the total variance in soil bacterial communities across two sampling months (Figure 4B). Among all the soil properties, soil ammonium, AP, and DOCN accounted for the greatest proportions of the variation in the bacterial communities (Figure 4B). There were significant positive relationships between soil moisture and Shannon index, as well as between soil DON and bacterial diversity indices (Chao1 and ACE) (Supplementary Table S4).

## Discussion

### Influence of Successive Rotation on Soil Bacterial and Fungal Community Diversity

In this study, both two sampling months saw a significant decline in richness and diversity of bacterial and fungal communities in TRP compared with those in FRP and SRP, but no significant difference was observed between FRP and SRP (Table 2). Our results partly agreed with a previous study that revealed the continuous decrease in bacterial diversity with successive rotation age, but the fungal diversity was more enriched under SRP and TRP compared with FRP (Wu et al., 2017). Conversely, other researchers reported a decrease in fungal diversity in consecutive monoculture of *C. equisetifolia* plantations, which was consistent with our results (Zhou et al., 2019). The contradictory results of successive rotations on fungal diversity were likely to be attributed to the different methods applied in these studies. Wu et al. (2017) utilized phospholipid fatty acids (PLFA) to assess the variations of soil fungal community, while Zhou et al. (2019) employed the pyrosequencing technique. Although PLFA is a rapid and quantitative analyses in determining soil microbial structure, next-generation sequencing appears to be more reliable to explore microbial community because it can deliver information at higher quantitative resolution and is not limited to only a few microbial groups such as PLFA. Biodiversity has been long recognized as a key factor in regulating ecosystem functioning; however, the importance of below-ground biodiversity is frequently underestimated due to their high redundancy. There are mounting evidence showing that the loss of soil microbial diversity would impair multiple ecosystem functions such as decomposition, nutrient cycling, and retention (van der Heijden et al., 1998; Griffiths et al., 2000; Wagg et al., 2014). Past observations have demonstrated that a greater diversity of soil organisms can enhance litter break down, reduce nutrient leaching losses, and maintain resource turnover between above- and below-ground communities (Wardle et al., 2004; van der Heijden et al., 2008). Majority of previous work has attributed the productivity decline in Chinese fir plantation to soil nutrient depletion (Ma et al., 2000; Yang et al., 2004), but this phenomenon cannot be simply solved by artificial fertilizing (Wu et al., 2017), which suggests the microorganisms may play a significant role in this problem. Our results confirmed the above hypothesis by exhibiting significant decrease in both fungal and bacterial richness and diversity in TRP compared with SRP (Tables 2, 3). Surprisingly, in most cases, no significant decline or even increase in soil C and N content were detected in SRP and TRP compared with FRP (Table 1). However, the consistent decrease in soil AP in SRP and TRP was observed in both sampling months compared with FRP (Table 1). It is, therefore, likely that soil P is a more limiting nutrient than C and N in subtropical forest soils in southern China, considering its deficiency in availability in Chinese fir plantation (Zou et al., 2015).

### Changes in the Composition of Soil Bacterial and Fungal Communities

In the present study, the results of deep sequencing demonstrated that *Basidiomycota*, *Ascomycota*, and *Mortierellomycota* were the predominant fungal phyla, while *Acidobacteria*, *Proteobacteria*, *Actinobacteria*, and *Chloroflexi* were the predominant bacterial phyla in the acidic forest soil, which was consistent with previous reports (Buée et al., 2009; Nie et al., 2018; Wang et al., 2018). Our results showed that the relative abundance of major fungal phyla (*Basidiomycota*, *Ascomycota*, *Mucoromycota*) was significantly changed by the successive rotations (Figure 1). Among them, *Mucoromycota* belong to the first colonizers followed by *Ascomycota* (Torres et al., 2005), which are considered as cellulose decomposers or sugar fungi with limited ability to degrade lignin (Osono, 2007). *Basidiomycota*, however, prevails in the later litter decomposition process as they are able to degrade recalcitrant lignin-containing material (Osono, 2007; Lundell et al., 2010).

The litterfall of Chinese fir plantation have unique morphological (toughness and needle-shaped) and chemical (high content of recalcitrant lignin) properties (Zhou et al., 2015), resulting in the slowness and difficulty in their decomposition and thus in nutrient return. In this case, fungi play an essential role in the organic matter decomposition and further C cycling in forest ecosystem for their ability to attack a wide spectrum of C, from labile to recalcitrant (Voříšková and Baldrian, 2013). The lowest relative abundance of *Ascomycota* and *Mucoromycota*, and the highest relative abundance of *Basidiomycota* were observed in TRP (Figure 1), indicating a possible shift in C composition at the different plantations with time. Specifically, with the increase in plantation generation, the more recalcitrant C was accumulated in soil, thus leading to the increase in lignin decomposer (*Basidiomycota*) and decrease in sugar fungi (*Ascomycota* and *Mucoromycota*). These results implied that successive planting of *C. lanceolata* plantations may gradually deplete the available C source in the soil, which was supported by the decline in soil DOC in TRP (Table 1). These data appeared to be in accordance with earlier findings that revealed a decrease in catabolic activity of soil microbial community in consecutive monoculture of Chinese fir plantations, following an order of FRP > SRP > TRP (Wu et al., 2017).

Previous studies have reported a high dominance of *Acidobacteria* (around 50%) in strongly acidic soil (4 < pH < 5) (Lauber et al., 2009), which was in good agreement with our results. The relative abundance of *Acidobacteria* ranked first among all bacterial species in our study, ranging from 45 to 57% (Figure 1). Different successive rotations had no significant effect on the relative abundance of major bacterial phyla (*Acidobacteria*, *Proteobacteria*, *Actinobacteria*), which is likely to be explained by the narrow range of pH change (approximately 0.3 unit) detected among different rotations (Table 1). This idea is supported by earlier research that demonstrated pH as the best predictor for bacterial community structure by surveying 88 soils across America (Lauber et al., 2009). No change in the relative abundance of *Acidobacteria* and *Actinobacteria* is unexpected because the former can degrade various polysaccharides including cellulose and xylan (Ward et al., 2009), while the latter are capable of utilizing lignin-derived compounds and, thus, are involved in the later stage of litter decomposition (Kirby, 2006). The possible explanation may be that fungal groups more actively participate in the organic matter decomposition, rather than the bacterial members, since the fungal community could better adapt to the nutrient-poor environment (Wu et al., 2017).

It is noteworthy that a large part of the sequenced ITS regions belonged to the unclassified fungi (12.6–31.6% and 40.6–87.1% at the phylum and genus level, respectively) due to incompletely annotated environmental samples. The inadequate taxonomic annotation and errors in taxonomic assignments of ITS sequences deposited in the international DNA database greatly limited our identification of the fungal species (Vilgalys, 2003). By contrast, more pertinent taxonomic information was provided for bacterial species at the phylum level (2.0–4.4%) but not at the genus level (69.1–73.5%).

### Environmental Factors Influencing Soil Microbial Community Structure

As demonstrated by RDA and correlation analysis (Figure 4 and Supplementary Table S4), the environmental changes that occur with different successive plantations contributed differently to the changes in fungal and bacterial communities. Generally, the shifts in fungal and bacterial communities were closely related to soil NH_4_^+^–N, DOC, pH, and AP, but with different directions and magnitudes for fungal and bacterial communities, respectively (Figure 4).

Soil DOC and ammonium played vital roles in driving the microbial community composition. Soil DOC is considered to be the most available source of C substrate for microbial population, and its correlation to microbial community has been reported extensively (Cookson et al., 2007). Comparatively, N is one of the most important nutrients for life, and NH_4_^+^–N accounts for one of the most easily accessible N source for microbes (He et al., 2017). Although soil pH has been viewed as a good predictor of bacterial community composition by multiple studies (Lauber et al., 2009), such a relationship was not as strong as the other factors (e.g. NH_4_^+^–N, DOC) observed in our study, which could be attributed to the narrow pH range (4.00–4.42) observed here.

## Conclusion

In summary, this study clearly demonstrated a significant reduction in soil bacterial and fungal community diversity in TRP compared with that of FRP and SRP in the Chinese fir plantation of southern China. Our results showed that successive rotations significantly changed the relative abundance of dominate phyla for the fungal community, but not the bacterial community. A shift in prevalence from phylum *Basidiomycota* to *Ascomycota* was observed in the third rotation plantation of Chinese fir, indicating the depletion of labile C and accumulation of more recalcitrant C in the soils under successive rotation of plantation. We also found that soil NH_4_^+^–N, pH, and DOC made considerable contributions in shaping fungal communities, while soil NH_4_^+^–N, DOCN, and AP primarily drive the changes in bacterial communities. This study represents a step toward insightful understanding of microbial communities in continuous monoculture plantations of Chinese fir.

## Data Availability Statement

The datasets generated for this study can be found in the PRJEB29419.

## Author Contributions

XL, YW, YL, HC, and YH contributed to conception and design of the study. YW, YL, and HC carried out the field work. XL organized the database and wrote the first draft of the manuscript. XL and YW performed the statistical analysis. All authors contributed to the manuscript revision, read and approved the submitted version. The corresponding author takes primary responsibility for communication with the journal and editorial office during publication.

## Conflict of Interest

The authors declare that the research was conducted in the absence of any commercial or financial relationships that could be construed as a potential conflict of interest.

## Footnotes

1 http://cutadapt.readthedocs.io/en/stable/

2 http://www.drive5.com/usearch/manual/uchime_algo.html

3 http://drive5.com/uparse/

## References

[B1] Bending G. D. Turner M. K. Rayns F. Marx M. C. Wood M. (2004). Microbial and biochemical soil quality indicators and their potential for differentiating arrears under contrasting agricultural management regimes. *Soil Biol. Biochem.* 36 1785–1792. 10.1016/j.soilbio.2004.04.035

[B2] Bennett A. J. Bending G. D. Chandler D. Hilton S. Mills P. (2012). Meetingthe demand for crop production: the challenge of yield declinein crops grown in short rotations. *Biol. Rev. Camb. Philos. Soc.* 87 52–71. 10.1111/j.1469-185x.2011.00184 21631700

[B3] Buée M. Reich M. Murat C. Morin E. Nilsson R. H. Uroz S. (2009). 454 pyrosequencing analyses of forest soils reveal an unexpectedly high fungal diversity. *New Phytol.* 184 449–456. 10.1111/j.1469-8137.2009.03003.x 19703112

[B4] Chen F. L. Zheng H. Zhang K. Ouyang Z. Y. Li H. L. Wu B. (2013). Soil microbial community structure and function responses to successive planting of *Eucalyptus*. *J. Environ. Sci.* 25 2102–2111. 10.1016/s1001-0742(12)60319-2 24494498

[B5] Chen G. S. Yang Z. J. Gao R. Xie J. S. Guo J. F. Huang Z. Q. (2013). Carbonstorage in a chronosequence of Chinese fir plantations in southern China. *Forest Ecol. Manage.* 300 68–76. 10.1016/j.foreco.2012.07.046

[B6] Cookson W. R. Osman M. Marschner P. Abaye D. A. Clark I. Murphy D. V. (2007). Controls on soil nitrogen cycling and microbial community composition across land use and incubation temperature. *Soil Biol. Biochem.* 39 744–756. 10.1016/j.soilbio.2006.09.022

[B7] DeForest J. L. Scott L. G. (2010). Available organic soil phosphorus has an important influence on microbial community composition. *Soil Sci. Soc. Am. J.* 74 2059–2066. 10.1371/journal.pone.0066184 23776630 PMC3679006

[B8] Edgar R. C. (2013). UPARSE: highly accurate OTU sequences from microbial amplicon reads. *Nat. Methods* 10 996–998. 10.1038/nmeth.2604 23955772

[B9] Edgar R. C. Haas J. C. Clemente J. C. Quince C. Knight R. (2011). UCHIME improves sensitivity and speed of chimera detection. *Bioinformatics* 27 2194–2200. 10.1093/bioinformatics/btr381 21700674 PMC3150044

[B10] Evans J. (1999). Sustainability of plantation forestry: impact of species changeand successive rotations of pine in the Usustu Forest, Swaziland. *S. Afr. For. J.* 84 63–70. 10.1080/10295925.1999.9631213

[B11] FAO (2006). *Global Forest Resource Assessment 2005.* Rome: Food and AgriculturalOrganization of the United Nations.

[B12] Griffiths B. S. Ritz K. Bardgett R. D. Cook R. Christensen S. Ekelund F. (2000). Ecosystem response of pasture soil communities to fumigation-induced microbialdiversity reductions: An examination of the biodiversity-ecosystem function relationship. *Okios* 90 279–294. 10.1034/j.1600-0706.2000.900208.x

[B13] Haas B. J. Gevers D. Earl A. M. Feldgarden M. (2011). Chimeric 16S rRNA sequence formation and detection in Sanger and 454-pyrosequenced PCR amplicons. *Genome Res.* 21 494–504. 10.1101/gr.112730.110 21212162 PMC3044863

[B14] Hartmann M. Howes C. G. Vanlnsberghe D. Yu H. Bachar D. Christen R. (2012). Significant and persistent impact of timber harvesting on soil microbial communities in Northern coniferous forests. *ISME J.* 6 2199–2218. 10.1038/ismej.2012.84 22855212 PMC3504969

[B15] He H. Chen X. J. Hou F. J. Wu Y. P. Cheng Y. X. (2017). Bacterial and fungal community structures in Loess Plateau grasslands with different grazing intensities. *Front. Microbiol.* 8:606. 10.3389/fmicb.2017.00606 28439265 PMC5383705

[B16] Huang Z. Q. Liao L. Cao C. (2000). Allelopathy of phenolics from decomposing stump-roots in replant Chinese fir woodland. *J. Chem. Ecol.* 26 2211–2219. 11767591

[B17] Jenkinson D. (1988). “Determination of microbial carbon and nitrogen in soil,” in *Advances in Nitrogen Cycling in Agriculture Ecosystems*, ed. Wilson J. (CAB International: Wallingfors), 368–386.

[B18] Kirby R. (2006). Actinomycetes and lignin degradation. *Adv. Appl. Microbiol.* 58 125–168. 10.1016/s0065-2164(05)58004-3 16509445

[B19] Lauber C. L. Hamady M. Knight R. Fierer N. (2009). Pyrosequencing-based assessment of soil pH as a predictor of soil bacterial community structure at the continental scale. *Appl. Envir. Microbiol.* 75 5111–5120. 10.1128/AEM.00335-09 19502440 PMC2725504

[B20] Liao J. Q. Wang J. Huang Y. (2015). Bacteiral community features are shaped by geographic location, physiocochemical properties, and oil contamination of soil in main oil fields of China. *Microb. Ecol.* 70 380–389. 10.1007/s00248-015-0572-0 25676171

[B21] Lin C. F. Yang Y. S. Chen G. S. Guo J. F. (2008). Decomposition dynamics of fine roots of *Cunninghamia lanceolata* in subtropics. *J. Subtrop. Resour. Environ.* 3 15–23. 10.13287/j.1001-9332.201807.003 30039659

[B22] Liu L. Duan Z. H. Xu M. K. Hu J. C. Wang S. L. Hu Z. G. (2010). Effect of monospecific and mixed *Cunninghamia lanceolata* plantations on microbial community and two functionalgenes involved in nitrogen cycling. *Plant Soil* 327 413–428. 10.1007/s11104-009-0067-1

[B23] Lundell T. K. Makela M. R. Hilden K. (2010). Lignin-modifying enzymes in filamentous basidiomycetes – ecological, functional and phylogenetic review. *J. Basic Microbiol.* 50 5–20. 10.1002/jobm.200900338 20175122

[B24] Ma X. Q. Heal K. V. Liu A. Q. Jarvis P. G. (2007). Nutrient cycling anddistribution in different-aged plantations of Chinese fir insouthern China. *For. Ecol. Manag.* 243 61–74. 10.1016/j.foreco.2007.02.018

[B25] Ma X. Q. Liu A. Q. Ma Z. Fan S. H. (2000). A comparative study on nutrient accumulation and distribution of different generations of Chinese fir plantations. *Chin. J. Appl. Ecol.* 11 501–506. 11767665

[B26] Martin M. (2011). Cutadapt removes adapter sequences from high-throughput sequencing reads. *EMBnet J.* 17:10 10.14806/ej.17.1.200

[B27] McNabb K. L. Wadouski L. H. (1999). Multiple rotation yields for intensivelymanaged plantations in the Amazon basin. *New For.* 18 5–15.

[B28] Moller J. Miller M. Kjoller A. (1999). Fungal–bacterialinteraction on beech leaves: influence on decompositionand dissolved organic carbon quality. *Soil Biol. Biochem.* 31 367–374. 10.1016/s0038-0717(98)00138-2

[B29] Nie Y. Wang M. Zhang W. Ni Z. Hashidoko Y. Shen W. (2018). Ammonium nitrogen content is a dominant predictor of bacterial community composition in an acidic forest soil with exogenous nitrogen enrichment. *Sci. Total Environ.* 624 407–415. 10.1016/j.scitotenv.2017.12.142 29262382

[B30] Osono T. (2007). Ecology of ligninolytic fungi associated with leaf litter decomposition. *Ecol. Res.* 22 955–974. 10.1007/s11284-007-0390-z 16917528

[B31] Selvaraj S. Duraisamy V. Huang Z. J. Guo F. T. Ma X. Q. (2017). Influence of long-term successive rotations and stand age of Chinese fir (*Cunninghamia lanceolata*) plantations on soil properties. *Geoderma* 306 127–134. 10.1016/j.geoderma.2017.07.014

[B32] Smith J. L. Paul E. A. (1990). “The significance of soil microbial biomass estimations,” in *Soil Biochemistry*, Vol. 6 eds Bollag J. M. Stotzky G. (New York, NY: CRC Press), 357–396.

[B33] Tian D. Xiang W. Chen X. Yan W. Fang X. Kang W. (2011). A long-term evaluation of biomass production infirst and secondrotations of Chinesefir plantations at the same site. *Forestry* 84 411–418. 10.1093/forestry/cpr029

[B34] Torres P. A. Abril A. B. Bucher E. H. (2005). Microbial succession in litter decomposition in the semi-arid Chaco woodland. *Soil Biol. Biochem.* 37 49–54. 10.1016/j.soilbio.2004.04.042

[B35] van der Heijden M. G. A. Bardgett R. D. van Straalen N. M. (2008). The unseen majority: Soil microbes as drivers of plant diversity and productivity in terrestrial ecosystems. *Ecol. Lett.* 11 296–310. 10.1111/j.1461-0248.2007.01139.x 18047587

[B36] van der Heijden Marcel G. A. Klironomos J. N. Ursic M. Moutoglis P. Streitwolf-Engel R. (1998). Mycorrhizal fungal diversity determines plant biodiversity, ecosystem variability and productivity. *Nature* 396 69–72. 10.1038/23932

[B37] Vance E. D. Brooks P. C. Jenkinson D. S. (1987). An extraction method for measuring soil microbial biomass C. *Soil Biol. Biochem.* 19 703–707. 10.1016/0038-0717(87)90052-6

[B38] Vilgalys R. (2003). Taxonomic misidentification in public DNA databases. *New Phytol.* 160 4–5. 10.1046/j.1469-8137.2003.00894.x 33873532

[B39] Voříšková J. Baldrian P. (2013). Fungal community on decomposing leaf litter undergoes rapid successional changes. *ISME J.* 7 477–486. 10.1038/ismej.2012.116 23051693 PMC3578564

[B40] Voříšková J. Brabcová V. Cajthaml T. Baldrian P. (2014). Seasonal dynamics of fungal communities in a temperate oak forest soil. *New Phytol.* 201 269–278. 10.1111/nph.12481 24010995

[B41] Wagg C. Bender S. F. Widmer F. van der Heijden M. G. A. (2014). Soil biodiversity and soil community composition determine ecosystem multifunctionality. *PNAS* 111 5266–5270. 10.1073/pnas.1320054111 24639507 PMC3986181

[B42] Wang Q. Wang C. Yu W. W. Turak Ali Chen D. W. Huang Y. (2018). Effects of nitrogen and phosphorus inputs on soil bacterial abundance, diversity, and community composition in Chinese fir plantations. *Front. Microbiol.* 9:1543. 10.3389/fmicb.2018.01543 30072961 PMC6060263

[B43] Wang Q. Wang S. Gao H. Yu X. (2005). Dynamics of soil activeorganic matter in Chinese fir plantations. *J. Appl. Ecol.* 16 1270–1274. 16252865

[B44] Ward N. L. Challacombe J. F. Janssen P. H. Henrissat B. Coutinho P. M. Wu M. (2009). Three genomes from the phylum Acidobacteria provide insights into the lifestyles of these microorganism in soils. *Appl. Environ. Microbiol.* 75 2046–2056. 10.1128/aem.02294-08 19201974 PMC2663196

[B45] Wardle D. A. Bardgett R. D. Klironomos J. N. Setälä H. van der Putten W. H. Wall D. H. (2004). Ecological linkages between aboveground and belowground biota. *Science* 304 1629–1633. 10.1126/science.1094875 15192218

[B46] Wu Z. Y. Li J. J. Zheng J. Liu J. F. Liu S. Y. Lin W. X. (2017). Soil microbial community structure and catabolic activity are significantly degenerated in successive rotations of Chinese fir plantations. *Sci. Rep.* 7:6691. 10.1038/s41598-017-06768-x 28751741 PMC5532251

[B47] Yang Y. S. Liu C. J. Kutsch W. Chen G. S. Yu X. T. (2004). Impact of continuous Chinese Fir monoculture on soil. *Pedosphere* 14 117–124.

[B48] Zhang J. Wang F. Che R. X. Wang P. Liu H. K. Ji B. M. (2016). Precipitation shapes communities of arbuscular mycorrhizal fungi in Tibetan alpine steppe. *Sci. Rep.* 6:23488. 10.1038/srep23488 PMC4802204 27002188

[B49] Zhou L. L. Shalom A. D. Wu P. F. Li S. B. Jia Y. Y. Ma X. Q. (2015). Litterfall production and nutrient return in different-aged Chinese fir (*Cunninghamia lanceolata*) plantations in South China. *J. For. Res.* 26 79–89. 10.1007/s11676-014-0011-y

[B50] Zhou L. T. Li J. J. Luo Y. Liu S. Y. Wang J. Y. Bai Y. (2019). Variation in soil fungal community structure during successive rotations of Casuarina equisetifolia plantations as determined by high-throughput sequencing analysis. *Plant Growth Regul.* 87 445–453. 10.1007/s10725-019-00483-5

[B51] Zou X. H. Wu P. F. Chen N. L. Wang P. Ma X. Q. (2015). Chinese fir root response to spatial and temporal heterogeneity of phosphorus availability in the soil. *Can. J. For. Res.* 45 402–410. 10.1139/cjfr-2014-0384

