# Supplementary material for: Response of Bacterial and Fungal Soil Communities to Chinese Fir (Cunninghamia lanceolate) Long-Term Monoculture Plantations
Source: Front Microbiol. 2020 Feb 28;11:181. doi: 10.3389/fmicb.2020.00181 (PMC7058989; doi:10.3389/fmicb.2020.00181)
Supplement: Supplementary file 1 [file Data_Sheet_1.doc]

**Supplementary Materials**

**Table S1** *P* values from two-way ANOVA (with repeated measure) of effects of planting rotations (R), sampling time (T) and their interaction (R×T) on soil physiochemical properties at the Xinkou Forest Farm, Fujian, China.

| Source | pH | | moisture | | TC | | TN | | TCN | | DOC | | DON | | DOCN | | MBC | | MBN | | MBCN | | | NH_4_^+^-N | | NO_3_^-^-N | | AP | |
| --- | --- | --- | --- | --- | --- | --- | --- | --- | --- | --- | --- | --- | --- | --- | --- | --- | --- | --- | --- | --- | --- | --- | --- | --- | --- | --- | --- | --- | --- |
| R | <0.001  *** | 0.002  ** | | 0.013  * | | 0.018  *** | | 0.14 | | 0.008  ** | | 0.44 | | 0.11 | | 0.13 | | 0.07 | | 0.45 | | | <0.001  *** | | <0.001  *** | | 0.032  * | |  |
| T | <0.001  *** | <0.001  *** | | 0.013  * | | 0.011*** | | 0.26 | | <0.001*** | | <0.001*** | | <0.001  *** | | <0.001  *** | | <0.001  *** | | | | 0.001  ** | 0.001  ** | | <0.001  *** | | <0.001  *** | |  |
| R×T | 0.072 | 0.004  ** | | 0.012  * | | 0.012  * | | 0.36 | | 0.024  * | | 0.20 | | 0.37 | | 0.07 | | 0.018  * | | 0.72 | | | <0.001  *** | | <0.001  *** | | 0.37 | |  |

Abbreviations: TC, total carbon; TN, total nitrogen; TCN, TC/TN ratio; DOC, dissolved organic carbon; DON, dissolved organic nitrogen; DOCN, DOC/DON ratio; MBC, microbial biomass carbon; MBN, microbial biomass nitrogen; MBCN, MBC/MBN ratio; AP, available phosphorus.

Significance levels: **P* < 0.05, ***P* < 0.01, ****P* < 0.001.

**Table S2** Total raw and final sequence numbers for each plantation rotations at Xinkou Forest Farm, Fujian, China.

| Season | Treatment | Sample | Total Raw 16S *r*RNA sequences | Clean 16S *r*RNA sequences | OTUs  3%cut-off | Total Raw ITS sequences | Clean ITS sequences | OTUs  3%cut-off |
| --- | --- | --- | --- | --- | --- | --- | --- | --- |
| December | NF | DecNF1 | 61136 | 57390 | 1284 | 81917 | 80096 | 1333 |
|  |  | DecNF2 | 86105 | 80860 | 1422 | 82635 | 80127 | 1207 |
|  |  | DecNF3 | 83587 | 80143 | 1387 | 83600 | 80059 | 1406 |
|  |  | DecNF4 | 85610 | 79646 | 1359 | 81384 | 80262 | 1354 |
|  | FRP | DecFRP1 | 84078 | 79576 | 1323 | 81503 | 80208 | 1137 |
|  |  | DecFRP2 | 88557 | 80102 | 1526 | 81898 | 80167 | 1651 |
|  |  | DecFRP3 | 93990 | 90384 | 1306 | 80963 | 80204 | 1302 |
|  |  | DecFRP4 | 91959 | 89526 | 1243 | 81795 | 80411 | 1337 |
|  | SRP | DecSRP1 | 82470 | 80090 | 1606 | 81556 | 80251 | 1497 |
|  |  | DecSRP2 | 81943 | 80174 | 1350 | 81201 | 80062 | 1435 |
|  |  | DecSRP3 | 82549 | 78093 | 1318 | 81469 | 80076 | 1464 |
|  |  | DecSRP4 | 69233 | 65880 | 1263 | 81409 | 80017 | 1322 |
|  | TRP | DecTRP1 | 58330 | 55856 | 1044 | 81822 | 80118 | 1237 |
|  |  | DecTRP2 | 68207 | 64242 | 1260 | 81523 | 80308 | 1189 |
|  |  | DecTRP3 | 84292 | 80379 | 1254  1213 | 80866 | 80198 | 1148 |
|  |  | DecTRP4 | 62848 | 59924 | 1213 | 87480 | 80188 | 1109 |
| June | NF | JunNF1 | 85661 | 80182 | 1355 | 82008 | 80319 | 1257 |
|  |  | JunNF2 | 77896 | 71827 | 1274 | 81682 | 80168 | 1164 |
|  |  | JunNF3 | 88644 | 85582 | 1192 | 82283 | 80193 | 1111 |
|  |  | JunNF4 | 93586 | 85626 | 1211 | 81561 | 80040 | 1225 |
|  | FRP | JunFRP1 | 87774 | 80083 | 1219 | 81397 | 80032 | 1327 |
|  |  | JunFRP2 | 82981 | 80339 | 1241 | 81383 | 80112 | 1123 |
|  |  | JunFRP3 | 82328 | 80166 | 1350 | 82961 | 80354 | 1390 |
|  |  | JunFRP4 | 82579 | 80204 | 1386 | 82092 | 80409 | 1498 |
|  | SRP | JunSRP1 | 90143 | 84215 | 1343 | 82521 | 80170 | 1401 |
|  |  | JunSRP2 | 85609 | 80075 | 1278 | 83156 | 80068 | 1544 |
|  |  | JunSRP3 | 84036 | 80021 | 1280 | 81543 | 80248 | 1276 |
|  |  | JunSRP4 | 95929 | 92288 | 1350 | 81250 | 80112 | 1321 |
|  | TRP | JunTRP1 | 80556 | 76595 | 1230 | 81242 | 80166 | 1387 |
|  |  | JunTRP2 | 86885 | 82856 | 1282 | 82027 | 80401 | 1386 |
|  |  | JunTRP3 | 92440 | 87533 | 1210 | 82768 | 80278 | 1299 |
|  |  | JunTRP4 | 84882 | 80095 | 1239 | 82614 | 80091 | 1186 |

**^a^** Sample name for each treatment, Dec means December, Jun means June, NF, natural forest; FRP, first rotation plantation; SRP, second rotation plantation; TRP, third rotation plantation, and the number after plantation rotations means the plot number.

**Table S3** *P* values from two-way ANOVA (with repeated measure) of effects of planting rotations (R), sampling time (T) and their interaction (R×T) on soil fungal and bacterial diversity indices at the Xinkou Forest Farm, Fujian, China.

| Source | Fungi | | | | Bacteria | | | |
| --- | --- | --- | --- | --- | --- | --- | --- | --- |
|  | OTUs | Chao1 | ACE | Shannon | OTUs | Chao1 | ACE | Shannon |
| R | 0.02* | 0.25 | 0.11 | 0.02* | 0.03* | 0.04* | 0.06 | 0.04* |
| T | 0.77 | 0.42 | 0.51 | 0.44 | 0.23 | 0.02* | 0.02* | 0.06 |
| R×T | 0.27 | 0.57 | 0.44 | 0.47 | 0.49 | 0.39 | 0.49 | 0.01* |

OTUs, operational taxonomic units (97% similarity). Significance levels: **P* < 0.05, ***P* < 0.01, ****P* < 0.001.

**Table S4** Pearson correlations between soil chemical properties and alpha diversity indices of bacterial and fungal communities in December and June at the Xinkou Forest Farm, Fujian, China.

|  | indices | month | pH | soil moisture | TC | TN | TCN | NH_4_^+^-N | NO_3_^-^-N |
| --- | --- | --- | --- | --- | --- | --- | --- | --- | --- |
| Bacterial community | OTUs | December | 0.30 | 0.19 | -0.34 | -0.22 | -0.44 | 0.13 | -0.19 |
|  | Chao1 |  | 0.28 | 0.23 | -0.27 | -0.13 | -0.44 | 0.08 | -0.14 |
|  | ACE |  | 0.28 | 0.19 | -0.29 | -0.16 | -0.44 | 0.10 | -0.15 |
|  | Shannon index |  | 0.20 | 0.14 | -0.33 | -0.28 | -0.26 | 0.20 | -0.21 |
|  | OTUs | June | 0.12 | 0.37 | 0.01 | 0.01 | -0.04 | 0.44 | -0.20 |
|  | Chao1 |  | 0.29 | 0.13 | 0.03 | 0.05 | -0.04 | 0.29 | -0.07 |
|  | ACE |  | 0.26 | 0.16 | 0.05 | 0.09 | -0.07 | 0.30 | -0.12 |
|  | Shannon index |  | -0.10 | **0.50** | 0.16 | 0.19 | 0.02 | 0.22 | 0.05 |
| Fungal community | OTUs | December | 0.30 | 0.28 | -0.17 | -0.0006 | -0.48 | 0.02 | -0.11 |
|  | Chao1 |  | 0.31 | 0.23 | -0.18 | -0.02 | -0.44 | 0.06 | -0.14 |
|  | ACE |  | 0.35 | 0.22 | -0.19 | -0.05 | -0.40 | 0.06 | -0.14 |
|  | Shannon index |  | 0.18 | 0.41 | 0.08 | 0.11 | -0.47 | -0.22 | -0.02 |
|  | OTUs | June | -0.23 | 0.25 | -0.22 | -0.26 | -0.07 | 0.10 | -0.19 |
|  | Chao1 |  | 0.07 | -0.09 | -0.22 | -0.20 | -0.17 | 0.12 | -0.36 |
|  | ACE |  | -0.02 | 0.01 | -0.23 | -0.24 | -0.14 | 0.12 | -0.29 |
|  | Shannon index |  | -0.19 | 0.43 | -0.26 | -0.26 | -0.17 | 0.19 | -0.18 |

to be continued

|  | indices | month | DOC | DON | DOCN | MBC | MBN | MBCN | AP |
| --- | --- | --- | --- | --- | --- | --- | --- | --- | --- |
| Bacterial community | OTUs | December | 0.22 | 0.48 | -0.48 | 0.46 | 0.38 | -0.13 | 0.17 |
|  | Chao1 |  | 0.10 | **0.57** | -0.45 | 0.41 | 0.36 | -0.18 | 0.22 |
|  | ACE |  | 0.05 | **0.54** | -0.48 | 0.42 | 0.37 | -0.16 | 0.22 |
|  | Shannon index |  | 0.08 | 0.44 | -0.46 | 0.22 | 0.06 | 0.02 | 0.22 |
|  | OTUs | June | -0.20 | -0.34 | -0.03 | -0.15 | -0.24 | 0.15 | 0.24 |
|  | Chao1 |  | -0.33 | -0.35 | -0.17 | -0.06 | -0.10 | 0.01 | 0.01 |
|  | ACE |  | -0.35 | -0.42 | -0.15 | 0.02 | -0.07 | 0.07 | 0.001 |
|  | Shannon index |  | 0.07 | -0.22 | 0.23 | -0.16 | -0.24 | 0.17 | 0.43 |
| Fungal community | OTUs | December | 0.17 | 0.26 | -0.03 | **0.78** | **0.77** | -0.35 | 0.11 |
|  | Chao1 |  | 0.13 | 0.24 | -0.07 | **0.78** | **0.76** | -0.33 | 0.18 |
|  | ACE |  | 0.11 | 0.23 | -0.07 | **0.78** | **0.74** | -0.29 | 0.16 |
|  | Shannon index |  | 0.35 | 0.32 | 0.20 | **0.65** | **0.75** | **-0.51** | -0.10 |
|  | OTUs | June | 0.12 | 0.007 | 0.15 | **-0.50** | -0.46 | 0.08 | -0.12 |
|  | Chao1 |  | -0.16 | -0.20 | -0.05 | -0.27 | -0.12 | -0.27 | -0.42 |
|  | ACE |  | -0.05 | -0.13 | 0.03 | -0.39 | -0.26 | -0.18 | -0.36 |
|  | Shannon index |  | 0.19 | 0.04 | 0.20 | -0.42 | -0.2 | 0.19 | 0.16 |

OTUs, operational taxonomic units (97% similarity). Bold values indicate a significant difference at *P* < 0.05. Abbreviation for soil chemical properties refers to Table 1.

**Figure S1** Relative abundance of the most abundant fungal (a) and bacterial (b) groups in different plantations in December at the phyla level. Others represented unclassified groups. Data are means ± standard error (n = 4). Significant differences are indicated with distinct letters. NF, natural forest; FRP, first rotation plantation; SRP, second rotation plantation; TRP, third rotation plantation.

**Figure S2** Relative abundance of the most abundant fungal (a) and bacterial (b) groups in different plantations in December at the genus level. Others represented unclassified groups. Data are means ± standard error (n = 4). Significant differences are indicated with distinct letters. NF, natural forest; FRP, first rotation plantation; SRP, second rotation plantation; TRP, third rotation plantation.
